# Ubiquitin-proteasome system regulation of bone remodeling in postmenopausal osteoporosis

**DOI:** 10.3389/fcell.2026.1788812

**Published:** 2026-04-20

**Authors:** Xiangqian Chen, Tong Zhao, Hailun Zhou, Qinghui Zeng, Hongkang Xu, Yulin Dai, Ming Yan

**Affiliations:** 1 Integrated Chinese and Western Medicine, Changchun University of Chinese Medicine, Changchun, China; 2 Department of Image Center, The Third Affiliated Hospital of Changchun University of Chinese Medicine, Changchun, China; 3 Northeast Asian Institute of Traditional Chinese Medicine, Changchun University of Chinese Medicine, Changchun, China; 4 Jilin Ginseng Academy, Changchun University of Chinese Medicine, Changchun, China; 5 School of Clinical Medicine, Changchun University of Chinese Medicine, Changchun, China

**Keywords:** bone metabolism, deubiquitinating enzymes, E3ligases, postmenopausal osteoporosis, ubiquitin-proteasome system

## Abstract

Postmenopausal osteoporosis (PMOP) is associated with declining estrogen levels, and this hormonal deficiency alters the physiological balance between bone formation and resorption. Substantial evidence indicates that protein homeostasis disorders play a significant role in the pathological process of PMOP. As the core system regulating protein homeostasis, the ubiquitin-proteasome system exerts crucial regulatory effects on bone metabolism under estrogen-deficient conditions. Abnormal activity of these ubiquitin-associated enzymes often leads to excessive degradation or abnormal accumulation of key regulatory proteins in bone metabolism, thereby exacerbating bone loss in PMOP. This review summarizes recent studies, focusing on how ubiquitin E3 ligases and deubiquitinating enzymes influence the function of osteoblasts and osteoclasts in PMOP. By synthesizing evidence from postmenopausal clinical samples and ovariectomized animal models, we further elucidate the specific roles of ubiquitin-related pathways in this disease. A deeper understanding of the mechanisms underlying UPS dysregulation in postmenopausal women holds promise for identifying novel molecular targets for future therapeutic strategies in PMOP.

## 1 Introduction

Osteoporosis (OP) is defined as a systemic skeletal disorder characterized by decreased bone mass, impaired bone microarchitecture, and increased bone fragility (Lorentzon and Cummings, 2015). The disease often presents with no obvious symptoms in its early stages. Most patients are only receiving a diagnosis after sustaining a low-energy fracture. Postmenopausal osteoporosis (PMOP) has become a major public health burden affecting the health of middle-aged and elderly women worldwide (Subarajan et al., 2024). Epidemiological studies indicate that approximately one-third of middle-aged and elderly women and one-fifth of men may experience osteoporotic fractures, with the incidence rate showing a persistent upward trend (Lorentzon et al., 2022). OP fractures in areas such as the spine and hips can lead to severe clinical consequences, including intense pain, loss of physical function, long-term disability, and even increased risk of mortality (Clynes et al., 2020). Therefore, developing effective interventions for postmenopausal osteoporosis is urgently needed.

**FIGURE 1:**
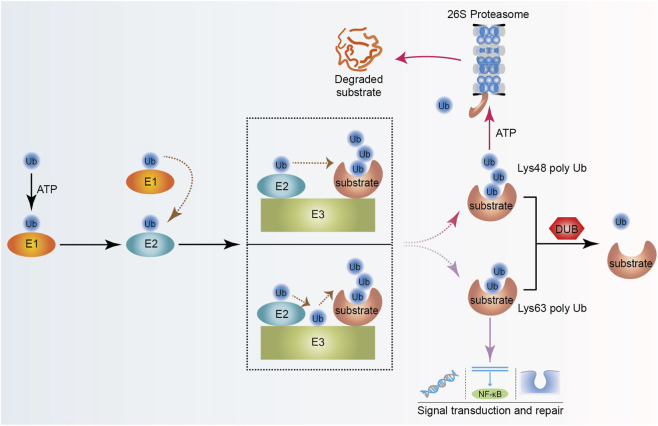
The ubiquitination and deubiquitination processes of proteins. With the participation of ATP, E1 catalyzes the activation of ubiquitin through a thioester bond; E2 obtains ubiquitin through thioester bond transfer; subsequently, E3 recognizes and transfers ubiquitin to the substrate; and finally, the K48-linked ubiquitinated substrate is degraded by the 26S proteasome, and the K63-linked ubiquitinated substrate participates in downstream signal transduction and repair. Alternatively, substrates that have completed ubiquitin labeling undergo a deubiquitination process in the presence of deubiquitinating enzymes. The detached ubiquitin reenters the ubiquitination cycle. Abbreviations: Ub, ubiquitin; E1, ubiquitin-activating enzyme; E2, ubiquitin-conjugating enzyme; E3, ubiquitin ligase; DUB, deubiquitinating enzyme.

**FIGURE 2:**
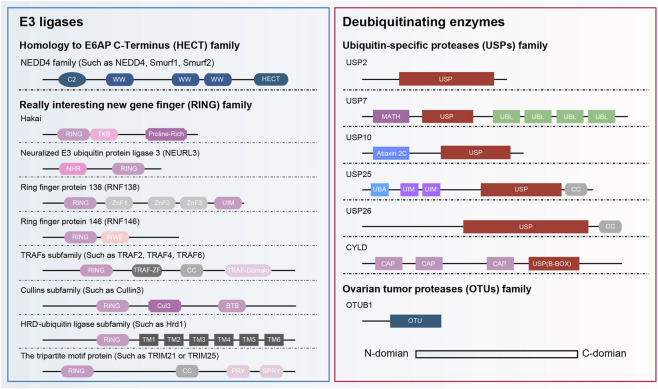
Protein domains of E3 ligases and deubiquitinating enzymes involved in PMOP regulation. E3 ligases primarily involve the HECT and RING families, while DUBs involve the USP and OTU families. Abbreviations: NHR, neuralized homology repeat; UIM, ubiquitin interaction motif; TRAF-ZF, TRAF-type zinc finger domain; CC, coiled-coil; PRY, PRY domain; SPRY, SPRY domain; BTB, bric-a-brac/tramtrack/broad-complex; MATH, meprin and TRAF homology domain; UBL, ubiquitin-like domain; UBA, ubiquitin-associated; CAP, cytoskeleton-associated protein-glycine-rich domains.

The ubiquitin-proteasome system (UPS) is central to maintaining protein homeostasis in cells. UPS is responsible for degrading more than 80% of functional or aberrantly expressed proteins in cells (Ravid and Hochstrasser, 2008). This system precisely regulates the differentiation, proliferation, and associated signaling networks of osteoblasts and osteoclasts, playing a crucial role in maintaining bone metabolic balance (Pan et al., 2022). Ubiquitination is a three-step, ATP-dependent enzymatic cascade reaction. The E3 ligase is crucial for recognizing target proteins, and its substrate specificity directly determines the biological effects of ubiquitin modification (Figure 1). According to catalytic-domain architecture, E3 ligases are divided into four major categories: HECT, RING, U-Box, and RBR types (Morreale and Walden, 2016; Yang et al., 2021). Deubiquitinating enzymes (DUBs) can specifically reverse ubiquitination modifications by removing ubiquitin or ubiquitin-like proteins from substrate proteins, thereby inhibiting protein degradation by the proteasome (Clague et al., 2019). Based on their catalytic domains, DUBs fall into seven evolutionarily conserved superfamilies: six (USP, OTU, UCH, MJD, ZUP1, and MINDY) are cysteine proteases, whereas the JAMM/MPN + family is the only metalloprotease-type group (Lange et al., 2022). This review summarizes the domain structures of the involved E3 ligases and DUBs (Figure 2).

Recent studies have revealed that PMOP caused by estrogen deficiency is accompanied by abnormal expression and dysfunction of E3 ligases and DUBs. Therefore, this review specifically focuses on the abnormal expression and function of E3 ligases and DUBs under estrogen deficiency conditions (studying OP samples from postmenopausal women or ovariectomy (OVX) animal OP models), establishing the connection between PMOP and UPS dysfunction. This review aims to provide a disease-contextualized perspective rather than a generic overview of ubiquitination in bone biology (Table 1).

**TABLE 1:** Effects of E3 ligases and deubiquitinating enzymes on the process of bone remodeling in postmenopausal osteoporosis.

Category	Family	Enzyme	Target or pathway	Evidence Model	Function	Therapeutic Relevance
E3 ligases	HECT	Nedd4	Runx1	Clinical samples; Human-BMSCs; OVX mice	Inhibition of osteogenic differentiation potential of BMSCs	Unexplored
​	​	​	Runx2	Mouse-BMSCs; OVX mice	​	Beraprost
​	​	​	p-p38	BMSCs; OVX mice	NEDD4 ubiquitinates p-p38, reduces its stability, and inhibits osteogenic differentiation of BMSCs	LncRNA-SNHG1
​	​	Smurf1, Smurf2	Smad	MC3T3-E1; OVX mice	Inhibition of osteogenic differentiation	Bortezomib
​	RING	Hakai	Smurf2	C3H10T1/2; OVX mice	Hakai increases Runx2 protein levels by degrading Smurf2, thereby positively regulating osteogenic differentiation	Unexplored
​	​	NEURL3	BMP7	Clinical samples; BMMs; OVX mice	Promote osteoclast differentiation	Unexplored
​	​	RNF138	Runx2	C3H10T1/2, rat calvarial osteoblast; OVX rat	Promote Runx2 degradation to inhibit osteoblast differentiation	Unexplored
​	​	RNF146	Axin	BMSCs; OVX mice	Enhances osteogenic differentiation	(DSS)_6_-ApoEVs^RNF146^
​	​	TRAF2	Gβl	Mouse-BMMs; OVX mice	TRAF2 promotes ubiquitinated degradation of Gβ1 and subsequently limits energy requirements for osteoclast differentiation via the PI3K-AKT pathway	TRAF1
​	​	TRAF4	Smurf2	Clinical samples; MSCs; OVX mice	Promote osteogenic differentiation of MSCs	Unexplored
​	​	TRAF6	​	BMMs; OVX mice	K48 linkage of TRAF6 mediates self-ubiquitination degradation to inhibit RANKL-induced osteoclast differentiation.	Curcumenol
​	​	​	​	RAW264.7, BMMs; OVX mice, OVX rat	K63-linked polyubiquitin chains of TRAF6 promote NF-κB/MAPK pathway activation for osteoclast differentiation.	*C. deserticola* ext-ract, Mogrol
​	​	Skp2	Runx2	MC3T3-E1; OVX rat	Impairment of osteoblast differentiation and mineralization	miR-495
​	​	Cullin3	Nrf2	RAW264.7; OVX rat	Cullin3 binds to Keap1 to inhibit Nrf2 signaling, thereby enhancing the production osteoclasts ROS and greatly promoting osteoporosis.	Icariin
​	​	TRIM21	TXNIP	BMMs; OVX mice	Trim21 deletion impaired osteoclast differentiation, thereby inhibiting bone resorption and maintaining bone mass to alleviate OVX-induced bone loss.	Unexplored
​	​	TRIM25	TREM1	Human-BMSCs, Human osteoblasts; OVX mice	TRIM25C promotes M2 macrophage polarization and osteogenic differentiation.	Unexplored
Deubiquitinating enzymes	USPs	USP2	LEF1	Clinical samples; Human-BMSCs; OVX mice	USP2 mediates deubiquitination of LEF1 to stabilize LEF1 protein, which in turn promotes osteogenic differentiation of BMSC.	Unexplored
​	​	USP7	HMGB1	Clinical samples; PBMCs, OVX mice	Promote osteoclast differentiation	P5091
​	​	​	TRAF6/ STING	Clinical samples; BMMs; OVX mice	Inhibits osteoclast differentiation and bone resorption	P5091
​	​	​	YAP1	BMSCs; OVX mice	Promotes bone formation by increasing osteoblast	Unexplored
​	​	USP10	NR3C1	Clinical samples; RAW264.7, BMMs, MC3T3-E1; OVX mice	Promotes osteoclast differentiation while inhibiting osteoblast differentiation	Unexplored
​	​	​	p53	MLO-Y4, MC3T3-E1; OVX mice	USP10 deubiquitinates and stabilizes p53, accelerating osteocyte and osteoblast senescence and bone loss	Spautin-1
​	​	USP25	Unexplored	Clinical samples	Further mechanism verification is needed	Unexplored
​	​	USP26	β-catenin	MSCs; OVX mice	Promotes osteogenic activity of MSCs	Unexplored
​	​	​	IκBα	BMMs; OVX mice	Impairment of osteoclast differentiation of BMMs	Unexplored
​	​	CYLD	WNK1	BMSCs; OVX mice	Inducing osteogenic differentiation of BMSCs	Unexplored
​	​	​	TRAF6	Clinical samples; BMMs; OVX mice	Inhibit osteoclastogenesis	miR-301-b
​	OTUs	OTUB1	Smurf1	Mouse-osteoblasts, BMSCs; OVX mice	OTUB1 attenuates Smurf1-mediated FGFR2 ubiquitination by inhibiting the interaction between Smurf1 and ubiquitin-conjugating enzyme in bones, thereby positively regulating osteogenic differentiation and mineralization	Unexplored

Abbreviations: HECT, homology to E6AP C-Terminus; RING, really interesting new gene finger; Nedd4, neuronal precursor cell-expressed developmentally downregulated 4; OVX, ovariectomy; Smurf, smad ubiquitination regulatory factor; Runx, runt-related transcription factor; BMSCs, bone marrow-derived mesenchymal stem cells; TRAF, TNF receptor-associated factor; RANKL, receptor activator of nuclear factor-κB ligand; NEURL3, neuralized E3 ubiquitin protein ligase 3; BMP7, bone morphogenetic protein 7; RNF, ring finger protein; TRIM, tripartite motif; LEF1, lymphoid enhancer binding factor 1; HMGB1, high mobility group box 1; PBMCs, peripheral blood mononuclear cells; Nrf2, nuclear factor erythroid 2-related factor 2; USPs, ubiquitin-specific proteases; STING, stimulator of interferon gene; YAP1, yes-associated protein 1; CYLD, cylindromatosis; OTUB1, ovarian tumor domain-containing ubiquitin aldehyde-binding protein 1.

## 2 Central role of estrogen in bone reconstruction

Bone is a continuously renewing organ that undergoes constant remodeling through the coordinated action of multiple cell lineages (Eriksen, 2010). Estrogen maintains bone homeostasis by dual regulation of bone metabolism (Emmanuelle et al., 2021). When estrogen is deficient, bone resorption accelerates relatively while bone formation lags relatively, resulting in net bone loss (Garnero et al., 1996). As a core regulator of bone remodeling, a sudden decline in estrogen is strongly correlated with rapid postmenopausal bone density loss (Khosla et al., 2012).

### 2.1 Direct effects of estrogen on bone formatio

The process of bone formation is highly complex, encompassing osteoblast differentiation, osteoid synthesis, and mineralization. Mesenchymal stem cells (MSCs) differentiate into osteoblasts. Mature osteoblasts subsequently undergo osteoid synthesis and bone matrix mineralization. The above processes are precisely regulated by signaling pathways such as Wnt/β-catenin and BMP/Smad (Cao and Chen, 2005; Gao et al., 2023). Estrogen is a key regulator in these pathways. After binding to ERα, estrogen promotes the translocation of β-catenin into the cell nucleus. This process activates the Wnt signaling pathway while simultaneously enhancing the activity of the BMP/Smad pathway, ultimately driving MSCs toward differentiation into the osteoblast lineage (Gao et al., 2013).

Transforming growth factor beta (TGF-β) is an indispensable factor in regulating osteogenesis, and its signaling pathway can influence the fate of MSCs (Wei et al., 2024). Studies have shown that TGF-β can recruit MSCs to the site of bone defects and promote the ability of MSCs to differentiate into the osteogenic lineage at physiological concentrations but inhibits this process at high physiological concentrations, showing dose-dependent bidirectional regulatory characteristics (Asparuhova et al., 2023). Estrogen upregulates TGF-β expression *in vitro*, thereby reducing osteoclast activity and promoting osteoclast apoptosis (Hughes et al., 1996).

Osteocytes constitute over 90% of adult bone cells and function as mechanosensors that regulate bone remodeling (Florencio-Silva et al., 2015; Qin et al., 2020). Estrogen–ERα signaling influences osteoblast-mediated bone-trabecular formation by modulating osteocyte responses to mechanical loading through the Wnt/β-catenin pathway (Armstrong et al., 2007; Kondoh et al., 2014). In addition, estrogen inhibits osteoblast and osteocyte apoptosis via rapid activation of the Src/Shc/ERK cascade (Kousteni et al., 2001; Kousteni et al., 2003).

### 2.2 Direct effects of estrogen on bone resorption

Osteoclasts are derived from multinuclear giant cells formed by the aggregation of monocytes/macrophages. They develop and adhere to the bone matrix and secrete acid and lytic enzymes to degrade bone tissue (Boyle et al., 2003). Osteoclasts are the primary effectors of bone resorption. Estrogen directly or indirectly regulates osteoclast differentiation, activation, and apoptosis at different levels.

Studies indicate that the effect of estrogen on osteoclasts is mediated through the osteoclast’s own receptor, ERα (Martin-Millan et al., 2010). During the proliferation and differentiation of osteoclasts, macrophage colony-stimulating factor (M-CSF) and receptor activator of NF-κB (RANK) ligand (RANKL) play key roles. M-CSF, tumor necrosis factor-alpha (TNF-α), and RANKL promote osteoclast survival by inhibiting mTOR/S6 kinase signaling (Glantschnig et al., 2003). Estrogen blocks M-CSF/RANKL-induced AP-1 transcription and inhibits RANKL-induced osteoclast differentiation by downregulating c-Jun expression (Shevde et al., 2000).

As a core regulator of bone resorption, RANKL initiates cascade signaling by binding to RANK (expressed on the osteoclast surface) to promote osteoclast differentiation, fusion, and proliferation (Ono et al., 2020). Specifically, upon RANKL stimulation, RANK induces the TRAF6-mediated assembly of K63-linked ubiquitin chains, which sequentially activate the NF-κB and MAPK signaling pathways (Thummuri et al., 2015). These pathways converge at the AP-1 (c-Fos/Jun) transcriptional complex, leading to the upregulation of NFATc1, a master regulator of osteoclastogenesis (Asagiri et al., 2005). Studies have shown that estrogen suppresses RANKL production by osteoblast lineage cells and lymphocytes (T cells and B cells) (Eghbali-Fatourechi et al., 2003). In the absence of estrogen, RANKL expression is induced. In addition, estrogen upregulates the expression of osteoprotegerin, which is known to be a decoy receptor that can compete with RANK for binding to RANKL, thereby reducing osteoclast differentiation (Bord et al., 2003).

Fas/FasL, a TNF-receptor-superfamily member, mediates apoptosis (Wallach et al., 1999). Estrogen induces osteoclast apoptosis through both autocrine and paracrine Fas/FasL mechanisms. Autocrinely, estrogen–ERα signaling upregulates FasL in osteoclasts of trabecular bone, promoting self-apoptosis (Nakamura et al., 2007). In the paracrine mechanism, estrogen upregulates FasL expression in osteoblasts (but not osteoclasts) through ERα, inducing the apoptosis of preosteoclasts and thereby preventing the formation of mature osteoclasts (Krum et al., 2008). Overall, estrogen regulates the lifespan of osteoclasts by inducing the Fas/FasL system, thereby maintaining normal bone mass.

### 2.3 Indirect effects of estrogen on bone remodeling

Beyond its direct actions on bone remodeling, estrogen also exerts indirect effects on skeletal homeostasis through immune-mediated modulation of pro-inflammatory cytokines. Cytokines such as IL-1, TNF-α, and IL-6 are key stimulators of osteoclast proliferation and differentiation, accelerating bone resorption and bone loss (Azuma et al., 2000; Kudo et al., 2002; Kudo et al., 2003). TNF-α also inhibits osteogenesis. TNF-α impedes the osteogenic differentiation of MSCs, reduces bone matrix production and mineralization, and promotes osteoblast apoptosis (Sun et al., 2016; Du et al., 2018). Under physiological conditions, estrogen effectively suppresses the production of IL-1, TNF-α, and IL-6, thereby maintaining skeletal homeostasis (Rogers and Eastell, 2001). When estrogen levels are insufficient, the expression of IL-6 and TNF-α increases, subsequently stimulating enhanced bone resorption (Abildgaard et al., 2020).

Menopause and the aging process are often accompanied by an imbalance in redox homeostasis. Reduced estrogen expression typically leads to elevated levels of reactive oxygen species (ROS) and increased oxidative stress (Zhou et al., 2016). Oxidative stress products such as ROS can inhibit osteoblast differentiation and promote osteoblast apoptosis, while also inducing osteoclast differentiation and enhancing bone resorption activity (Arai et al., 2007; Almeida et al., 2010; Agidigbi and Kim, 2019). Estrogen counteracts oxidative stress-induced bone loss by acting as an antioxidant and upregulating the expression of antioxidant enzymes (Almeida et al., 2010; Yang et al., 2023).

## 3 Involvement of E3 ligases

Ubiquitination is a dynamic and widely present posttranslational modification pathway, the main function of which is to mediate protein hydrolysis. An increasing number of studies support that ubiquitination plays a crucial role in the development of PMOP. Dysregulated ubiquitination disturbs the fine balance between bone resorption and formation, leading to abnormal remodeling under estrogen-deficient conditions. Among the enzymes that mediate this process, E3 ligases orchestrate the selective degradation, stabilization, or activation of key regulators involved in osteoblast and osteoclast biology. This section summarizes the emerging mechanisms by which different classes of E3 ligases, focusing primarily on HECT- and RING-type proteases, contribute to the development and progression of PMOP.

### 3.1 The NEDD4 subfamily

Neuronal precursor cell-expressed developmentally downregulated 4 (Nedd4), the largest subfamily of HECT-type E3 ligases, was initially identified as a regulator of central nervous system development in mice (Harvey and Kumar, 1999). Recent studies have revealed dual mechanisms through which Nedd4 disturbs RUNX protein homeostasis. On the one hand, in PMOP patients, Nedd4 mediates the degradation of Runx1 through ubiquitination (Huang et al., 2023). Specifically, in the bone microenvironment of patients with PMOP, GATA4 (a pioneer factor of ERα), MALAT1, and KHSRP were downregulated, whereas Nedd4 is aberrantly overexpressed. Correspondingly, the overexpression of GATA4 significantly activated the transcription of the lncRNA MALAT1, which binds KHSRP to form a complex to degrade Nedd4 mRNA to inhibit the ubiquitination degradation of Runx1 by Nedd4, ultimately promoting the osteogenic differentiation of MCSs and improving PMOP (Huang et al., 2023). On the other hand, Nedd4 inhibits the osteogenic differentiation potential of BMSCs by down-regulating Runx2 expression through ubiquitination, whereas beraprost can inhibit p53 signaling to counteract this process, significantly enhancing the osteogenic capacity in PMOP models (Zheng et al., 2021). Additionally, Nedd4 ubiquitinates p-p38, reducing its stability and thus negatively regulating osteogenic differentiation of BMSCs, which contributes to PMOP progression (Jiang et al., 2019).

The E3 ligases Smad ubiquitination regulators 1 and 2 (Smurf1 and Smurf2) are two members of the Nedd4 family that influence bone metabolic homeostasis by negatively regulating the TGF-β/BMP signaling pathway (Wang D. et al., 2023). Smurf1 regulates BMP signaling and inhibits osteogenic differentiation by promoting ubiquitin-dependent degradation of Smad1 and Smad5 (Zhang et al., 2017). Unlike Smurf1, Smurf2 inhibits osteoblast-dependent osteoclastogenesis by mono-ubiquitinating Smad3 to block RANKL expression induced by Smad3 interaction with vitamin D (Xu et al., 2017). A recent study reports that bortezomib, a first-line drug for multiple myeloma, exerts a therapeutic effect in PMOP by restoring the balance of Smurf-mediated ubiquitination (Fang et al., 2021). Mechanism studies have revealed that bortezomib blocks ubiquitin-mediated Smad degradation by down-regulating expression of Smurf1 and Smurf2, thereby up-regulating Runx2 expression and promoting MC3T3-E1 differentiation into osteoblasts (Fang et al., 2021). The results indicate that bortezomib alleviates bone loss by promoting osteogenic differentiation in OVX mice.

### 3.2 Hakai

Hakai (CBLL1) was originally identified as an E3 ligase for E-cadherin (Escuder-Rodríguez et al., 2025). Recent studies indicate that Hakai also plays a role in broader biological functions beyond epithelial plasticity. A recent study identified Hakai as a novel Runx2-interacting protein. It counteracts Smurf2-mediated proteasomal degradation of Runx2 (Upadhyay et al., 2024a). Mechanistically, Hakai binds to Runx2 and promotes the degradation of Smurf2. This action stabilizes Runx2 protein levels and enhances its transcriptional activity. Functional experiments showed that overexpression of Hakai significantly promoted the differentiation and mineralization of C3H10T1/2 cells into osteoblasts. Knockdown of Hakai reduces Runx2 expression and impaires osteogenic activity. In an OVX rat model, the expression of Hakai and Runx2 in bone tissue was synchronously downregulated. This suggests that reduced Hakai levels may contribute to the pathological progression of PMOP. These findings suggest that Hakai acts as a positive regulator of bone formation and holds promise as a novel therapeutic target for PMOP.

### 3.3 NEURL3

Neuralized E3 ubiquitin protein ligase 3 (NEURL3) has recently been implicated in bone remodeling in PMOP. Analysis of serum samples from osteoporosis patients and OVX mouse models revealed significant upregulation of NEURL3 (Cheng et al., 2025). Experiments demonstrate that knocking down NEURL3 significantly inhibits osteoclast differentiation of BMMs. Mechanistic studies reveal that NEURL3 interacts with BMP7 and promotes its ubiquitin-dependent degradation, thereby facilitating osteoclast differentiation. Silencing BMP7 markedly reversed the inhibitory effect of NEURL3 knockdown on osteoclast differentiation. This indicates that the NEURL3-BMP7 pathway represents a key ubiquitin-dependent mechanism driving osteoclast generation in estrogen-deficient animal models. A limitation of this study is the lack of explicit emphasis on postmenopausal status in the clinical osteoporosis samples (Cheng et al., 2025).

### 3.4 Ring finger protein-type E3 ligase

RING finger (RNF) proteins are a class of important E3 ubiquitin ligases that directly catalyze the conjugation of ubiquitin to target proteins. Studies have found that RNF138 reduces the osteogenic differentiation capacity of C3H10T1/2 by promoting Runx2 ubiquitination and degradation (Upadhyay et al., 2024b). In bone tissue from OVX rat models, elevated levels of RNF138 were observed to correlate with reduced Runx2 expression. Deletion of RNF138 significantly increased Runx2 protein levels and enhanced osteogenic potential. This indicates that RNF138 can inhibit osteoblast differentiation. Abnormal upregulation of RNF138 may drive the progression of PMOP.

Unlike RNF138, RNF146 actively promotes osteogenic differentiation of BMSCs by regulating the Wnt/β-catenin signaling pathway. Previous studies have found that RNF146 ubiquitinates and degrades Axin, thereby stabilizing β-catenin and enhancing Wnt signaling (Nakamura et al., 1998; Zhang et al., 2011). Recently, engineered apoptotic extracellular vesicles (EVs) carrying RNF146 showed significant bone targeting and osteogenic effects in an estrogen-deficient OP mouse model (Gui et al., 2024). This demonstrates the advantages of E3 ligase-modified delivery systems in the treatment of PMOP. Due to differences in protein structure and target sites, RNF138 and RNF146 exert opposing effects on osteogenic differentiation. Selectively inhibiting negative regulators like RNF138 or enhancing positive regulators such as RNF146 offers a promising strategy for counteracting PMOP.

### 3.5 TRAFs subfamily

The TNF receptor-associated factor (TRAF) family is highly conserved phylogenetically. These scaffold proteins can connect the TNF receptor family and IL-1R/Toll receptor to downstream signaling pathways, thereby activating NF-κB and MAPK (Wajant et al., 2001). This family comprises seven members. Except for TRAF1, all other members possess an N-terminal RING domain. The RING structure confers E3 ubiquitin ligase activity upon them (Park, 2018). Therefore, TRAF1 does not have the ability to directly catalyze substrate ubiquitination. TRAF1 functions as a scaffold adaptor protein by regulating the activity of other E3 ligases within the TRAF family, particularly TRAF2. Recent studies have shown that TRAF1 and TRAF2 promote the progression of OP in postmenopausal animal models by promoting osteoclast differentiation of BMMs (Kang et al., 2025). Mechanistically, TRAF1 interacts with TRAF2 to inhibit TRAF2-mediated ubiquitination and degradation of Gβl, a core component of the mTORC2 complex. Stabilized Gβl enhances AKT phosphorylation, thereby increasing mitochondrial oxidative phosphorylation to meet the high energy demands of osteoclast differentiation through the PI3K-AKT signaling (Kang et al., 2025). However, it should be noted that direct *in vivo* evidence linking TRAF2 to PMOP is still limited, and further studies are needed to clarify its specific role under estrogen deficiency conditions.

TRAF4 can inhibit the progression of PMOP (Li et al., 2019). TRAF4 expression is significantly reduced in bone sections from PMOP patients and OVX rats. Further studies revealed that TRAF4 positively regulates MSC osteogenic differentiation by promoting ubiquitin-dependent degradation of Smurf2 through K48-linked chains. Previous studies have confirmed the important role of Smurf2 in regulating osteogenesis, which can ubiquitinate and degrade Smad2 and Runx2, thereby inhibiting osteogenesis (Zhang et al., 2001; Choi et al., 2014). Thus, the TRAF4–Smurf2 axis may constitute a positive regulatory loop that counteracts PMOP development.

TRAF6 is a pivotal E3 ligase in RANKL-mediated osteoclastogenesis, and its activity is tightly controlled by differential polyubiquitin linkages. K63-linked ubiquitination of TRAF6 stabilizes the protein and promotes the recruitment of downstream signaling complexes, thereby activating NF-κB and MAPK pathways that drive osteoclast differentiation and bone resorption. In contrast, K48-linked ubiquitination targets TRAF6 for proteasomal degradation, thereby terminating signaling and preventing excessive osteoclast activation. Recent studies demonstrated that promoting TRAF6 K48-linked ubiquitination, such as by curcumenol effectively suppresses osteoclast activity and prevents estrogen deficiency-induced bone loss, whereas inhibition of K63-linked ubiquitination such as by C. deserticola extract or mogrol similarly attenuates TRAF6 signaling and mitigates PMOP (Zhang et al., 2019; Wang et al., 2021; Chen et al., 2022). Collectively, different TRAF6 ubiquitination sites profoundly affect osteoclast differentiation, and identifying upstream regulators of TRAF6 ubiquitination opens a promising avenue for future research on PMOP therapeutics.

### 3.6 Skp2

S-phase kinase-associated protein 2 (Skp2) is a key F-box component of the SCF E3 ligase complex. SKP2 can target Runx2 to inhibit osteoblast differentiation of MC3T3-E1. Mechanistic studies have shown that Skp2 binds to the 104–340 amino acid region of Runx2, promoting Runx2 ubiquitination and proteasome degradation (Thacker et al., 2016). This ubiquitination significantly reduces Runx2 activity, thereby impairing osteoblast differentiation and mineralization. In OVX rats, Skp2 expression was significantly upregulated, and Runx2 expression was reduced. Estrogen replacement therapy restored Runx2 levels while reducing Skp2 expression. This study links Skp2 to the pathological mechanism of estrogen-mediated PMOP.

Skp2 expression is also regulated at the epigenetic level. In an OVX fracture model, lysine (K)-specific demethylase 5A (KDM5A) suppresses the transcription of miR-495. This suppression allows Skp2 to evade negative regulation by microRNA (Li et al., 2025). Upregulation of Skp2 levels promotes Runx2 ubiquitination and degradation. Under estrogen-deficient conditions, this process delays osteoblast differentiation and bone fracture healing. Interfering with KDM5A downregulates Skp2 expression, thereby increasing Runx2 protein levels. This measure improves bone mass, trabecular microstructure, and bone mechanical properties in OVX rats. The miR-495-Skp2 pathway regulation of PMOP opens a novel research avenue. Upstream epigenetic regulatory factors and downstream ubiquitination modifications work synergistically to precisely regulate osteoblast function under estrogen deficiency.

### 3.7 Cullin3

Cullin3 is the core scaffold component of the Cullin-RING family of E3 ubiquitin ligase. Cullin3 can assemble into a complex with the adaptor protein Keap1. This complex recognizes the transcription factor Nrf2, subsequently mediating its ubiquitination and degradation (Villeneuve et al., 2010). This process participates in the regulation of cellular redox homeostasis. Under physiological conditions, the body maintains redox homeostasis. In pathological contexts such as estrogen deficiency, persistent oxidative stress enhances Cullin3-mediated degradation of Nrf2, thereby impairing antioxidant defenses and favoring osteoclastogenesis. Recent pharmacological studies have also confirmed the importance of this pathway in the pathological progression of PMOP. For example, the natural flavonoid compound icariin has been shown to inhibit RAW264.7-induced osteoclastogenesis by suppressing Cullin3 expression to stabilize Nrf2 and reduce ROS accumulation, ultimately alleviating OP symptoms in OVX rats (Si et al., 2024).

In recent years, the role of Keap1 in osteoclast regulation has gradually attracted attention. Studies have shown that novel Keap1-Nrf2 protein interaction inhibitors (such as KCB-F06) can effectively prevent bone loss caused by OVX by blocking Keap1-mediated Nrf2 ubiquitination and maintaining the antioxidant defense function of osteoclasts (Chen et al., 2024). Similarly, the clinical-stage Nrf2 activator bitopertin suppresses osteoclast differentiation by disrupting Keap1–Nrf2 binding, reducing Nrf2 degradation, and engaging the novel Nrf2–iron–ornithine axis, thereby improving OVX-related bone loss (Dong et al., 2024). Although these pharmacological studies did not directly address Cullin3, it is well recognized that Keap1 requires Cullin3 binding to function as an E3 ligase, thereby mediating Nrf2 degradation and oxidative stress regulation. This mechanistic interdependence supports the view that Cullin3-Keap1 acts as a redox-sensitive E3 ligase connecting oxidative imbalance with osteoclast-driven bone loss in PMOP and thus represents a promising avenue for further investigation.

### 3.8 TRIM protein family

Members of the tripartite motif (TRIM) family of E3 ligases have emerged as important regulators of bone remodeling under estrogen-deficient conditions. TRIM21 has recently been implicated in the biological process of inhibiting osteoclast differentiation under conditions of estrogen deficiency (Peng et al., 2025). In TRIM21 conditional knockout mice subjected to OVX, TRIM21 deficiency significantly attenuates estrogen deficiency–induced bone loss. Proteomic profiling and immunoblotting analyses indicated that these effects are mediated via upregulation of thioredoxin-interacting protein (TXNIP) and modulation of the NOD-like receptor signaling pathway. Mechanistically, TRIM21 deletion upregulates TXNIP and suppresses RANKL-induced osteoclast differentiation via the NOD-like receptor pathway. Collectively, the TRIM21–TXNIP axis represents a novel mechanism implicated in PMOP and a promising target for therapeutic intervention.

Similarly, TRIM25 is involved in immune-bone crosstalk during PMOP. Mechanistically, TRIM25 promotes the ubiquitination and subsequent proteasomal degradation of triggering receptor expressed on myeloid cells-1 (TREM1), thereby favoring macrophage M2 polarization and attenuating proinflammatory signaling (Zhang et al., 2024). M2 macrophages primarily secrete anti-inflammatory cytokines, which help promote osteoblast differentiation and bone mineralization (Horwood, 2016). Overexpression of TREM1 significantly reversed TRIM25-mediated macrophage polarization and osteogenesis. Administration of TRIM25-rich EVs derived from BMSCs significantly reduces trabecular bone loss in OVX mice and improves micro-CT imaging parameters. Collectively, TRIM21 and TRIM25 are key E3 ligases regulating PMOP. Their mechanisms of action differ yet complement each other. TRIM21 primarily restricts osteoclast differentiation, while TRIM25 promotes osteoblast differentiation by regulating macrophage polarization.

## 4 DUBs regulate PMOP

E3 ligases attach polyubiquitin chains to target proteins via lysine residues. The 26S proteasome recognizes and degrades the ubiquitinated target proteins labeled by E3 ligases. Conversely, DUBs reverse the ubiquitination process by removing ubiquitin chains. This process enhances the stability and functional activity of the target proteins. In PMOP, DUBs act as key regulators, balancing estrogen deficiency-induced hyperubiquitination. This section summarizes recent research indicating that DUBs of the USP and OTU subfamilies participate in the regulation of bone metabolism through different molecular mechanisms.

### 4.1 USP2

Ubiquitin-specific protease 2 (USP2) participates in the regulation of various bone-related diseases, including osteosarcoma, osteoarthritis, and OP. For example, USP2 interacts with TRAF2 and promotes TRAF2 deubiquitination, thereby enhancing inflammatory responses in rheumatoid arthritis (Liu et al., 2025). Parathyroid hormone (PTH) is a drug approved by the U.S. FDA for the treatment of severe osteoporosis and related fractures. The efficacy of PTH is primarily based on its promotion of bone anabolism (Casado-Díaz et al., 2019). The process by which teriparatide (PTH1-34) regulates osteoblast proliferation has been found to correlate with the expression levels of USP2 (Shirakawa et al., 2016). At the molecular level, USP2 deubiquitinates and stabilizes the lymphoid enhancer binding factor 1 (LEF1) (Zhang Z. et al., 2025). Elevated LEF1 levels promote the proliferation and osteoblast differentiation of BMSCs. In an OVX mouse model, overexpression of USP2 effectively reverses bone loss and alleviates estrogen deficiency-induced PMOP symptoms.

### 4.2 USP7

USP7 shows promise as a novel therapeutic target for PMOP. USP7 bidirectionally regulates bone metabolism processes. On the one hand, USP7 can positively regulate osteoclast differentiation (Lin et al., 2023). In human CD14^+^ monocytes from osteoporotic patients, USP7 expression is elevated and promotes osteoclast differentiation by deubiquitinating and stabilizing high-mobility group box 1 (HMGB1). Pharmacologic inhibition with the USP7-specific inhibitor P5091 reduces HMGB1, suppresses osteoclastogenesis, and attenuates OVX-induced bone loss *in vivo*, indicating a pro-osteoclast role for USP7 in that context (Lin et al., 2023).

On the other hand, USP7 has emerged as an important negative regulator of osteoclast differentiation and bone resorption in both *in vitro* and *in vivo* models (Xie et al., 2023). In bone marrow-derived macrophages (BMMs) and RAW264.7 cell systems, USP7 suppresses osteoclastogenesis through two complementary mechanisms. First, it impairs K63-linked ubiquitination of TRAF6, thereby dampening RANKL-NF-κB/MAPK signaling (without degrading TRAF6). In addition, USP7 stabilizes stimulator of interferon genes (STING), which induces the production of interferon-β and cooperatively suppresses osteoclast differentiation alongside the TRAF6 pathway. Inhibition of USP7 accelerates osteoclast differentiation and bone resorption, whereas USP7 overexpression produces the opposite effect. *In vivo* experiments found that lentiviral-mediated overexpression of USP7 attenuates RANKL-induced calvarial bone loss, while USP7 knockdown exacerbates this bone loss. Importantly, reduced expression of USP7 has been observed in OVX mice and in cancellous bone samples from patients with OP, suggesting that USP7 downregulation may contribute to PMOP.

In addition to the osteoclast lineage, USP7 also plays a positive role in the osteoblast lineage (Wang X. et al., 2023). Studies show that BMSC-derived EVs increase bone mass in OVX mice while elevating USP7 levels in bone. Mechanistically, EV-carried USP7 stabilizes the yes-associated protein 1 (YAP1) through deubiquitination, enhances Wnt/β-catenin signaling, and promotes osteoblastogenesis.

The above evidence suggests that the multifaceted regulation of bone metabolism by USP7 may be related to its specific context-dependent nature, which varies according to cell type, substrate availability, and inflammatory status. Different USP7 substrates dominate in different cellular and microenvironments. For example, under inflammatory conditions characterized by elevated HMGB1 signaling, USP7 activity may preferentially promote osteoclastogenesis through stabilization of HMGB1. In contrast, under physiological or estrogen-deficient conditions, USP7 may limit osteoclast differentiation through modulation of TRAF6 ubiquitination and activation of the STING–IFN-β pathway. In addition, cell lineage differences can also affect bone metabolism processes, as USP7 can also promote osteoblast differentiation by stabilizing YAP1. Notably, the context-dependent regulatory behavior of USP7 is not restricted to bone metabolism but has also been widely reported in other biological processes, such as the regulation of apoptosis in cancer cells (Bhattacharya et al., 2018). Therefore, the selection of USP7 as a potential therapeutic target for PMOP should be cautious and consider the current cellular and inflammatory context.

### 4.3 USP10

USP10 appears to be involved in the regulation of bone homeostasis during the progression of PMOP. In a study investigating the role of nuclear receptor subfamily 3 group C member 1 (NR3C1) in OP, USP10 expression was found to be significantly elevated in bone tissues of PMOP patients compared with healthy controls, and a similar increase was observed in OVX mice (Zhou et al., 2024). Mechanistic analysis revealed that USP10 functions as a deubiquitinase to remove polyubiquitin chains from NR3C1, thereby stabilizing NR3C1. NR3C1 acts as a transcription factor to regulate transcriptional repression of CST3. CST3 encodes the protein cystatin C, which is involved in osteoclast differentiation in RAW264.7 cells and BMMs, while promoting osteogenic differentiation in MC3T3-E1 cells. Another report demonstrated that estrogen acts as a negative regulator of USP10 in in vitro and *in vivo* models relevant to the study of bone metabolism (Wei et al., 2021). USP10, which is directly linked to p53 regulation, was downregulated by estrogen, leading to accelerated p53 degradation, alleviation of senescence phenotypes in osteocytic and osteoblastic models, and restoration of bone mass in OVX mice. Conversely, USP10 overexpression abolished the protective effects of estrogen. Taken together, these studies highlight the possibility of USP10 as a potential therapeutic target for PMOP.

### 4.4 USP25

USP25 is expressed in peripheral blood mononuclear cells (PBMCs), and its expression levels are regulated by estrogen (Shen et al., 2021). The GSE56815 dataset, revealed that some USP family members (*USP1*, *USP7*, *USP9X*, *USP16* and *USP25*) were highly enriched in postmenopausal female samples. Western blot experiments revealed that the protein expression of USP25 in PBMCs from low-BMD samples was significantly greater than that in high-BMD samples and that the protein expression of USP25 in PBMC samples from postmenopausal women was significantly greater than that in samples from premenopausal women. These findings indicate that USP25 may be involved in regulating the pathological process of PMOP. In addition, the expression levels of USP25 in healthy whole blood and PBMC samples were highly positively correlated with the expression level of TRAF6. This suggests that USP25 may promote osteoclast differentiation by stabilizing TRAF6 protein, thereby participating in the bone loss process of PMOP. This conclusion requires further mechanistic research to confirm.

### 4.5 USP26

USP26 is a typical DUB, and past studies have focused on its relationship with male infertility (Liu et al., 2018). In the latest study, USP26 was shown play a bone-protective role by balancing bone resorption and bone formation (Li et al., 2022). The study revealed that USP26 was significantly downregulated in BMMs and MSCs from OVX mice, indicating that estrogen deficiency affects USP26 expression. In turn, supplementation with USP26 significantly reduced ovariectomy-induced bone loss. Mechanistic studies revealed that deubiquitination of USP26 reduced the degradation of IκBα, which in turn inhibited NF-κB-p65 activation, thereby preventing BMMs from differentiating into osteoclasts (Li et al., 2022). In addition, the authors reported that USP26 can increase the osteogenic activity of MSCs by stabilizing β-catenin with deubiquitination. Similarly, USP26 deficiency can impair the osteogenic differentiation of MSCs. In summary, USP26 is a potential therapeutic target for treating PMOP.

### 4.6 CYLD

Cylindromatosis (CYLD), a DUB associated with tumor suppression, has recently been identified as a core regulator of bone remodeling under estrogen deficiency conditions. In the bone tissue of OVX mice, CYLD expression is significantly reduced, accompanied by downregulation of With-no-K[Lys] kinase-1 (WNK1) and activation of the NLRP3 inflammasome. Restoration of CYLD or WNK1 expression promotes osteogenic differentiation of BMSCs and inhibits inflammasome-associated cytokines. Mechanistically, CYLD directly interacts with and deubiquitinates WNK1, thereby stabilizing its protein level and preventing proteasomal degradation (Jiang et al., 2024). Stabilized WNK1 inhibits NLRP3 inflammasome activation, reduces pyroptotic signaling, and enhances osteogenic differentiation. In addition, CYLD is involved in the regulation of osteoclasts (Liu et al., 2020; Zhu et al., 2020). It recruits TRAF6 and removes K63-linked polyubiquitin chains, thereby inhibiting NF-κB/MAPK signaling pathway activation and preventing osteoclast differentiation of BMMs. In conclusion, CYLD promotes osteoblast differentiation and inhibits osteoclastogenesis through different molecular pathways to maintain bone homeostasis, and stabilizing CYLD expression helps to alleviate PMOP.

### 4.7 OTUB1

OTU domain-containing ubiquitin aldehyde-binding protein 1 (OTUB1) is a member of the OTU family with a unique non-canonical deubiquitination mechanism. Previous studies have shown that OTUB1 performs a deubiquitination function to inhibit the degradation of active Smad2/3 to enhance TGF-β signaling, via a mechanism in which OTUB1 binds and inhibits E2, resulting in the inability of ubiquitin to be transferred to the E3 ligase (Juang et al., 2012). Recent studies have reported the role of OTUB1 in promoting OB bone formation. OTUB1 attenuates the ubiquitination-mediated degradation of fibroblast growth factor receptor 2 (FGFR2) by inhibiting the linkage of E2 to Smurf1 (Zhu et al., 2023). The mechanism is that OTUB1 binds to FGFR2 and interferes with the assembly of the SMURF1-UbcH5C complex, thereby preventing SMURF1-mediated ubiquitination and subsequent lysosomal degradation of FGFR2. Stabilized FGFR2 maintains downstream AKT and ERK signaling pathways, promotes osteogenic differentiation, and enhances bone matrix mineralization. In OVX mice, OTUB1 expression in bone tissue was significantly reduced, while local delivery of AAV9-OTUB1 restored bone volume, trabecular architecture, and osteogenic marker expression, confirming its protective role under estrogen deficiency. In summary, OTUB1 regulates bone remodeling by blocking ubiquitin transfer to inhibit ubiquitination, highlighting the unique mechanism and therapeutic potential of OTUB1 in regulating PMOP.

**FIGURE 3:**
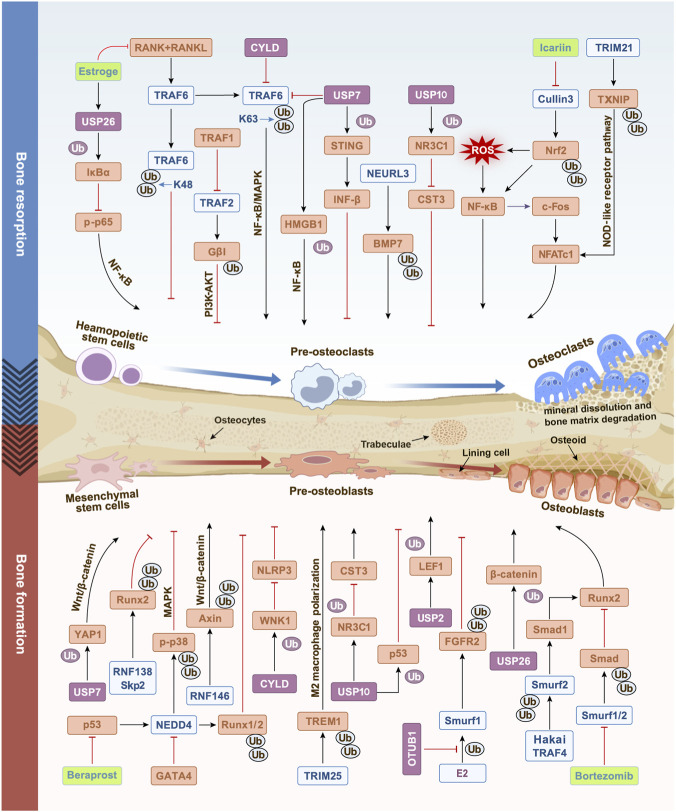
Mechanistic regulatory network of bone metabolism in postmenopausal osteoporosis (PMOP) by E3 ligases and deubiquitinating enzymes (DUBs). Mesenchymal stem cells differentiate into osteoblasts to regulate bone formation, while hematopoietic stem cells differentiate into osteoclasts to regulate bone resorption. In the estrogen-deficient PMOP model, an imbalance in the reversible ubiquitin degradation system of E3 ligases and DUBs modulates bone metabolic pathways through multiple mechanisms, thereby affecting the course of PMOP. Straight arrows indicate activation or positive regulation. T-shaped arrows indicate inhibition or negative regulation; blue icons represent E3 ligases; blue “Ub” labels indicate ubiquitination; purple icons represent DUBs; purple “Ub” labels indicate deubiquitination; green icons denote inhibitors, and orange icons indicate regulated cytokines.

Taken together, the aforementioned studies highlight the complex regulatory role of the UPS in bone metabolism during PMOP. At the molecular level, most E3 ligases associated with PMOP belong to the HECT and RING families, while DUBs are primarily concentrated in the USP and OTU families. These enzymes influence bone remodeling through various mechanisms and act on both the osteoblast and osteoclast lineages. For example, NEDD4 targets the ubiquitination and degradation of Runx1/2—key transcription factors for osteogenic gene expression—and p-p38, respectively, to inhibit osteoblast differentiation. USP10 jointly inhibits the differentiation of MC3T3-E1 into osteoblasts via the NR3C1 and p53 pathways. Conversely, some enzymes are involved in the regulation of the osteoclast lineage. For instance, TRAF6 maintains the NF-κB/MAPK signaling pathway via K63-linked ubiquitination to promote osteoclast differentiation, whereas K48-linked ubiquitination mediates its own degradation and contributes to the restoration of bone homeostasis. USP10 promotes osteoclast differentiation by deubiquitinating and stabilizing NR3C1. Furthermore, certain DUBs exhibit context-dependent functions. For instance, under inflammatory conditions, USP7 promotes osteoclast differentiation by stabilizing HMGB1, but it can also inhibit osteoclastogenesis by suppressing K63-linked ubiquitination of TRAF6. These findings suggest that E3 ligases and DUBs together form a complex regulatory network that maintains the balance of PMOP bone remodeling. The key regulatory relationships described in this review are summarized in Figure 3.

## 5 Conclusion and outlook

Estrogen deficiency triggers complex alterations in the bone remodeling process, which typically involves multiple signaling pathways. The evidence summarized in this review suggests that ubiquitin-mediated protein regulation disrupts bone metabolism through synergistic (and sometimes even antagonistic) effects on osteoblast and osteoclast differentiation, leading to postmenopausal bone loss. Ubiquitin E3 ligases and DUBs do not function independently but form a reversible regulatory network that modulates bone cell function in a context-dependent manner. Furthermore, most ubiquitin proteases reported in the review do not act directly on mature bone cells but rather affect the differentiation of precursor cell populations such as MSCs or osteoclast progenitor cells, thereby indirectly regulating the differentiation of osteoblasts and osteoclasts. We cannot simply define ubiquitin-related pathways as entirely beneficial or entirely detrimental. PMOP is more likely the result of a selective dysregulation of protein turnover mechanisms, disrupting the delicate balance between bone formation and resorption.

Existing research shows significant differences in the strength of evidence supporting different regulatory molecules. Some ubiquitin-related enzymes have been shown to be associated with bone loss in postmenopausal patient samples and ovariectomized animal models, while evidence for other enzymes is mainly based on *in vitro* experiments or animal models, lacking direct human sample data. This distinction warrants attention, as the differences between ubiquitin-mediated physiological bone metabolism regulation and disease-related bone loss may be misinterpreted. Therefore, these findings should be cautiously correlated with the pathological mechanisms of PMOP. Furthermore, although numerous studies suggest that various E3 ligases and DUBs are involved in the regulation of bone metabolism, such as RNF213, USP14, and USP25, which have been reported to be involved in regulating processes such as bone loss or osteogenic dysfunction, they lack systematic validation from patients or OVX animal models to prove a direct association with PMOP (Jin et al., 2025; Zhang Y. et al., 2025). Future research needs to establish more rigorous disease models to verify the direct causal relationship between E3 ligases and DUBs and PMOP.

In therapeutic strategies, ubiquitin-mediated protein homeostasis regulation has emerged as a common target for multiple experimental interventions in postmenopausal osteoporosis. Interventions utilizing bioactive small molecules, EVs-based delivery systems, AAV9-mediated gene transfer, and other nanomaterial- or biomaterial-based carriers have demonstrated the potential to precisely regulate ubiquitin-mediated homeostasis in bone tissue within PMOP models. This offers novel therapeutic avenues for PMOP beyond traditional hormone replacement therapy and anti-resorptive drugs.

In summary, estrogen deficiency alters the balance between ubiquitination and deubiquitination in bone tissue, thereby synergistically affecting the functions of osteoblasts and osteoclasts. Integrating research at different molecular levels into the specific pathological framework of PMOP helps explain why interventions targeting single pathways often have limited effectiveness, and also highlights the importance of viewing ubiquitin-mediated regulation as a dynamic network in postmenopausal osteoporosis.
